# Tryptophan Analogues with Fixed Side‐Chain Orientation: Expanding the Scope

**DOI:** 10.1002/cbic.202000424

**Published:** 2020-09-24

**Authors:** Lennart Nicke, Philip Horx, Ronny Müller, Sylvia Els‐Heindl, Armin Geyer

**Affiliations:** ^1^ Faculty of Chemistry Philipps-University Marburg Hans-Meerwein-Strasse 4 35032 Marburg Germany; ^2^ Faculty of Life Sciences Institute of Biochemistry Leipzig University Brüderstrasse 34 04103 Leipzig Germany

**Keywords:** ghrelin, metadynamics, NMR spectroscopy, peptide, tryptophan

## Abstract

A generalized synthetic strategy is proposed here for the synthesis of asymmetric β‐indoylated amino acids by 8‐aminoquinoline (8AQ)‐directed C(sp^3^)‐H functionalization of suitably protected precursors. Peptides containing one of the four stereoisomers of (indol‐3‐yl)‐3‐phenylalanine at position 2 of the parent peptide KwFwLL‐NH_2_ (w=d‐Trp) cover a wide range of activities as ghrelin receptor inverse agonists, among them the most active described until now. This application exemplarily shows how β‐indoylated amino acids can be used for the systematic variation of the position of an indole group in a bioactive peptide.

## Graphical abstract

**Immobile indole stays put**: Conformationally locked side‐chain rotamers of tryptophan were obtained by a general β‐indoylation method. The new type of β‐indoyl‐α‐amino acids allow systematic optimization of the β‐indole group orientation in different peptide environments. Four ghrelin receptor inverse agonists containing stereoisomeric β‐indoylated α‐amino acids span a broad range of activities.

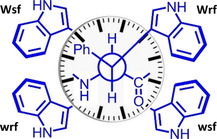

The strategic positioning of d‐amino acids,^[1]^ N‐methylation,^[2]^ and pseudo‐amide bonds^[3]^ are tools for the conformational design of bioactive peptides. These methods often intend to influence the conformational pattern of side chains but not their overall number. Significantly harsher conformational locks are local side‐chain‐to‐backbone cyclizations^[4]^ or bicyclic dipeptides^[5]^ that often go at the expense of side‐chain functional groups because the combination of local backbone rings and side‐chain functionality requires much synthetic effort^[6]^ or is restricted to hydroxy substituents.^[7]^ Instead of losing side‐chain functionality to achieve a severe conformational restriction, we recently proposed doubly functionalized asymmetric β‐branched amino acids as local conformational locks.^[8]^ Synthetic methods towards unsymmetrical β‐diaryl‐α‐amino acids are known for many aromatic groups^[9]^ but derivatives of tryptophan for solid‐phase peptide synthesis (SPPS) were not described before. Peptides containing stereoisomers of conformationally locked β‐indoylated α‐amino acids showed high or low receptor activities depending on whether the side‐chain orientation represented fit the bioactive conformation or not.^[10]^ Based on the preliminary results, we complete the set of stereoisomers of Trp/Phe diaryl amino acids here and expand the β‐indoylation protocol towards other DNA‐encoded α‐amino acids.

Tryptophan is found in numerous peptide hormones, peptide drugs, metabolites, and proteins, whereby the indole orientation plays a central role in transmitting hydrophobic, π–π and π‐cation interactions β‐methylated tryptophans were introduced into somatostatin analogues to investigate the bioactive side chain conformation.^[11]^ Exemplarily, the short ghrelin receptor inverse agonist KwFwLL‐NH_2_ is known to be binding with its aromatic core – wFw – to an aromatic region in the ghrelin receptor important for basal activity. By this interaction, the constitutive activity of the GPCR is reduced.^[12]^ The exchange of tryptophans for a synthetic amino acid like naphthylalanine is a gambling on biological activity often accompanied by reduced solubility of the peptide containing a hydrophobic 10π electron side chain and by unspecific receptor binding of the extended aromatic ring system. The β‐indoylated set of 20 encoded α‐amino acids proposed in Figure 1 moves the indole group around its natural position in a predicable “hopping” fashion to one of its neighboring amino acids along the sequence. This systematic structural variation differs from classical tryptophan mutations in several respects. Firstly, the orientation and mobility of the indole group is significantly more restricted than in Trp because β‐branched amino acids have preferred rotamers about *χ*1 and a restricted *syn*/*anti* equilibrium about *χ*2. Secondly, four different stereoisomers are accessible for the amino acids which bear a stereocenter on Cβ investigated below for the W/F chimeric β‐branched α‐amino acids Wrf, Wsf, wrf, and wsf (the first letter of the three‐letter code of the β‐indoylated amino acids stands for l‐ or d‐tryptophan (W or w), the second for either *R* or *S* stereochemistry of Cβ and the third letter for phenylalanine as defined in ref. [8]). Thirdly, unspecific stacking of the extended aromatic surface of asymmetric β‐diarylated amino acids is reduced because the two rings are inclined against each other and thus prevent simple edge‐on or side‐on π‐stacking that leads to high solubilities in spite of the large overall π‐surface. Finally, a common synthetic strategy allows for the β‐indoylation of most of the encoded amino acids with exceptions of already β‐branched α‐amino acids (I, V and T), which are more reactive on Cγ,^[13]^ and sulfur containing Met, Cys, or Gly, which lacks Cβ. In order to show that β‐indoylation is not restricted to benzylic groups, we included the synthesis and conformational analysis of β‐indoyl‐proline Wsp (Figure 1).

**Figure 1:**
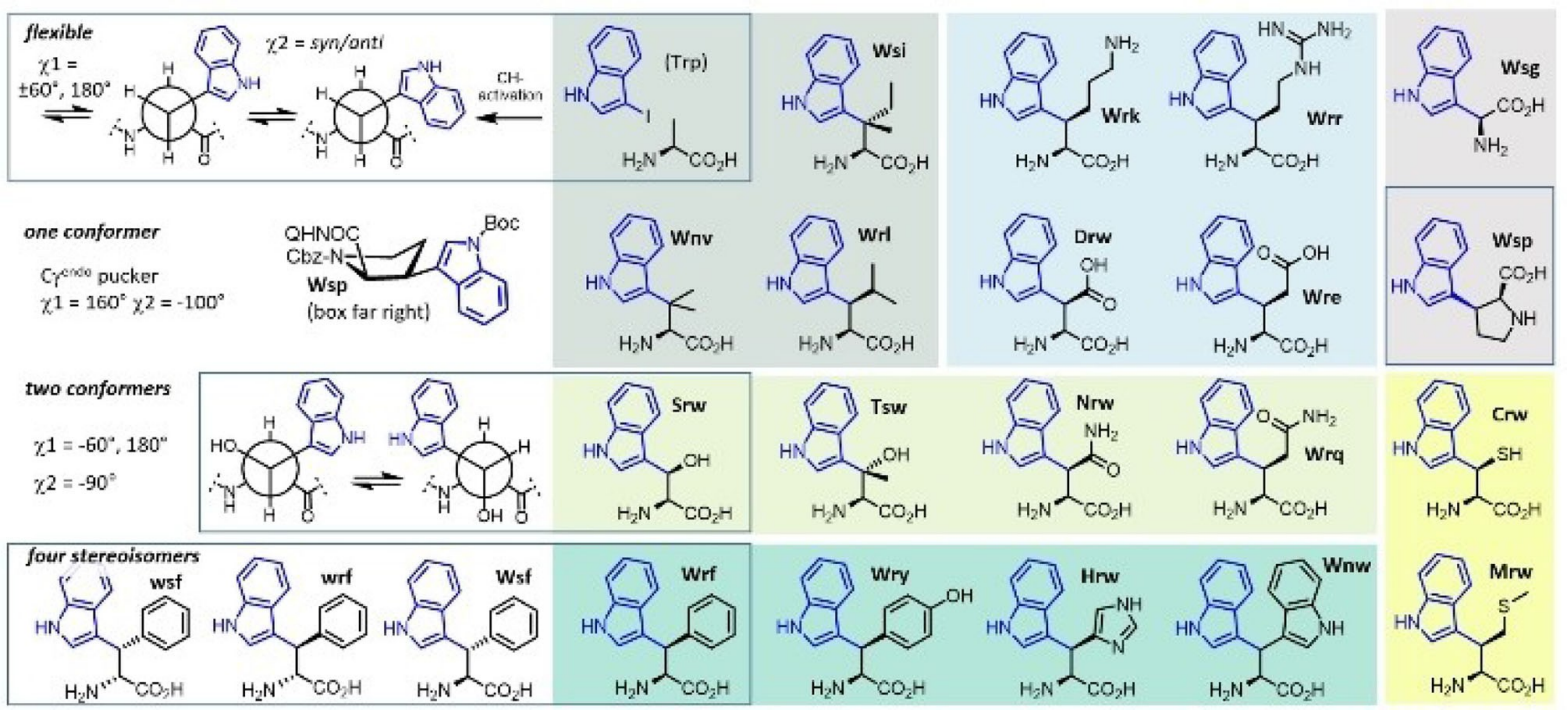
The β‐indoylated versions of 20 genetically encoded α‐amino acids are shown on colored backgrounds (aliphatic side chains, Pro, and Gly in different shades of grey, charged in light blue, polar in lime, aromatic in green and sulfur‐containing in yellow). Four representative examples are highlighted in boxes. β‐indoylation of Ala (upper left) yields Trp which can populate up to six conformers about *χ*1 (staggered rotamers in Newman plot) and *χ*2 (*syn/anti*). Wsp (2nd row) is the most restricted derivative occupying a preferred ring conformation and a single rotamer about *χ*1. The β‐hydroxylated tryptophan Wrs is characterized by the equilibrium between two rotamers about *χ*1 (3rd row). All four stereoisomers are shown for β‐indoylated F (4th row) to show the variability of CH activation in the cases where a stereocenter is formed at Cβ (all except for A, V, W and G). The n in Wnv and Wnw stands for no stereocenter at Cβ.

To access both d‐configured β‐phenyl‐tryptophans for standard Fmoc‐SPPS, our previously described method for sequential C−H activation and amide destabilization was followed^[8]^ starting from d‐Ala and d‐Phe (Scheme 1). Furthermore, the synthesis was streamlined by subjecting the crude activated amide directly to hydrolysis conditions after an aqueous workup for removal of excess triphosgene. The desired building blocks were obtained in optimal purity and gram‐scale quantities. The NMR‐spectra were in accordance with their respective enantiomers.

**Scheme 1:**
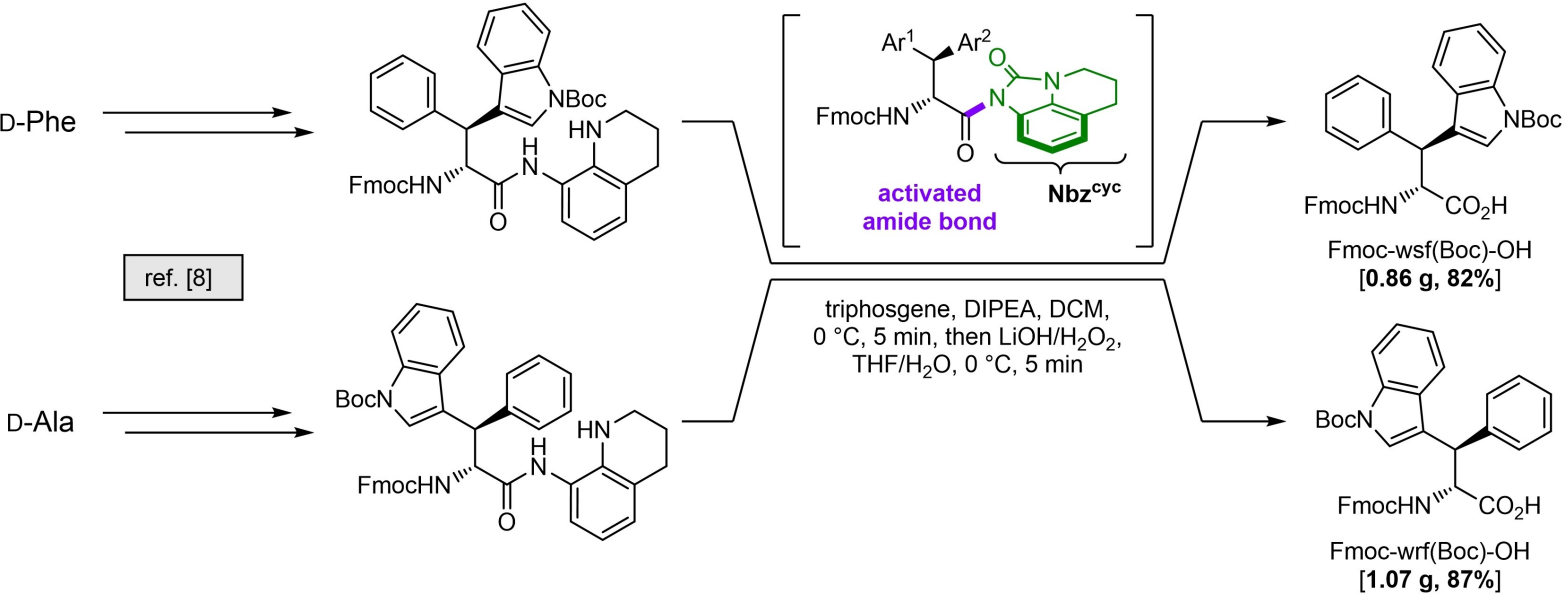
Synthesis of β‐indoylated Fmoc‐wsf(Boc)‐OH and Fmoc‐wrf(Boc)‐OH for peptide synthesis. Pd‐catalyzed CH activation is mediated by the 8‐aminoquinoline directing group. Transfer hydrogenation of the heterocyclic ring followed by acylation with triphosgene yields the strained acyl urea intermediate shown in green which is directly hydrolyzed to the free acids.

The four diaryl amino acids Wrf, Wsf, wrf and wsf were introduced to substitute d‐Trp^2^ in the ghrelin receptor inverse agonist KwFwLL‐NH_2_ (**4**). Peptide synthesis was carried out according to ref. [10] and synthetic data can be found in the Supporting Information. In this set of peptides, the two analogues Wrf^2^ (**5**) and Wsf^2^ (**6**) were already described and now, wrf^2^ (**7**) and wsf^2^ (**8**) were synthesized. The four peptides and two known inverse agonists were tested towards their activity on the ghrelin receptor that was stably transfected in COS7 cells and examined in the Cisbio IP‐One assay as described in the supplementary section. The activities are shown in Figure 2 and the EC_50_ values are listed in Table 1. Notably, both analogues wrf^2^ (**7**) and wsf^2^ (**8**) showed high inverse agonist efficacy, comparable to KbFwLL‐NH_2_ (**3**, b=d‐3‐benzothienyl alanine), which is one of the most active inverse agonists derived from substance P.^[14]^ Additionally, wsf^2^ (**8**) possessed an activity slightly more active than the d‐Bth^2^ analogue **3** with 23.1 nM in the IP‐One accumulation assay. In contrast, Wrf^2^ (**5**) and Wsf^2^ (**6**) had EC_50_ values in the high nanomolar range and of the two, only Wsf^2^ was able to fully decrease the constitutive activity of the ghrelin receptor at high concentrations. These experiments demonstrate the high activity range inducible by diaryl amino acids that only differ in the orientation of tryptophan and phenylalanine. Although physical parameters such as hydrophobicity and size are similar, up to 20‐fold activity differences could be observed. Further combinations to enhance activity are possible due to the wide range of indoylated amino acids that can be accessed by the established synthesis protocols.

**Figure 2:**
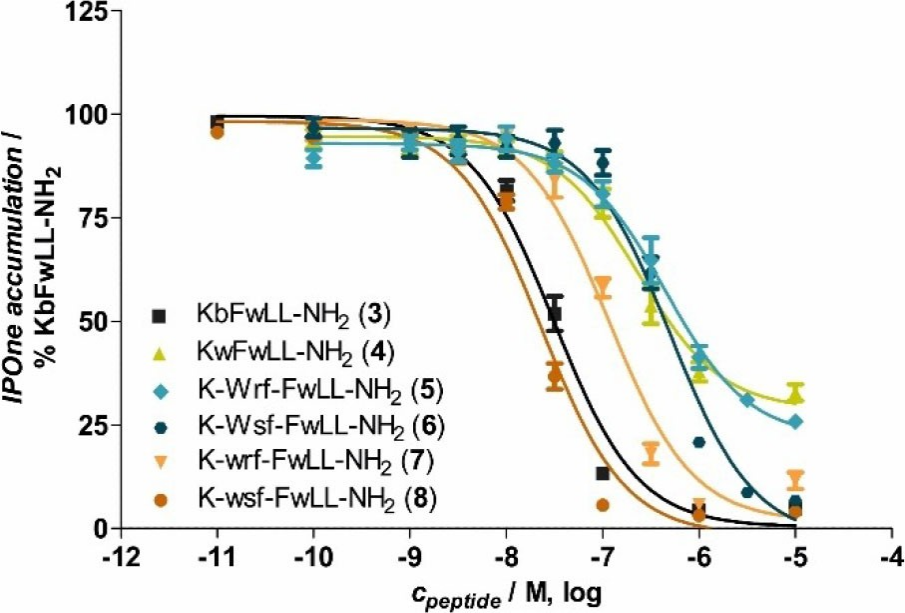
Activity data from the IP‐One assay. The assay was carried out in COS7 cells that were stably transfected with the ghrelin receptor. Data are the mean ±SEM of at least two independent experiments performed in triplicate. The orientation of the phenyl and indole side chains noticeably influences the inverse agonist activity of the analogues at the ghrelin receptor.

**Table 1:** IP‐One assay from ghrelin receptor inverse agonist analogues. pEC_50_ and *E*_max_ values are the mean±SEM of at least two independent experiments; *E*_max_ is normalized to KbFwLL‐NH_2_ (**3**).

	Peptide	EC_50_ [nM]	pEC_50_±SEM	*E*_max_±SEM [Δ%]
**3**	KbFwLL‐NH_2_	31.2	7.51±0.03	99±2
**4**	KwFwLL‐NH_2_	232	6.63±0.07	66±3
**5**	K‐Wrf‐FwLL‐NH_2_	445	6.35±0.07	71±3
**6**	K‐Wsf‐FwLL‐NH_2_	499	6.30±0.06	99±4
**7**	K‐wrf‐FwLL‐NH_2_	115	6.94±0.07	97±4
**8**	K‐wsf‐FwLL‐NH_2_	23.1	7.64±0.05	100±3

Each of the four Trp‐derivatives Wrf, Wsf, wrf and wsf is locked in a different rotamer about both side‐chain torsions *χ*1 (N‐Cα‐Cβ‐C3^ind^) and *χ*2(Cα‐Cβ‐C3^ind^‐C2^ind^). The large ^*3*^ *J* coupling between Hα and Hβ and the short distance between Hα and H2^ind^ are a common structural feature of the four amino acids. This predictable conformational preference is transferred to the peptide of interest. Drawing the amino terminus on the left‐hand side shows how the indole group moves along four quadrants of the Newman plot from 10 o′clock (Wsf) along 2 and 4 to 8 o′clock in wsf (Figure 3).

**Figure 3:**
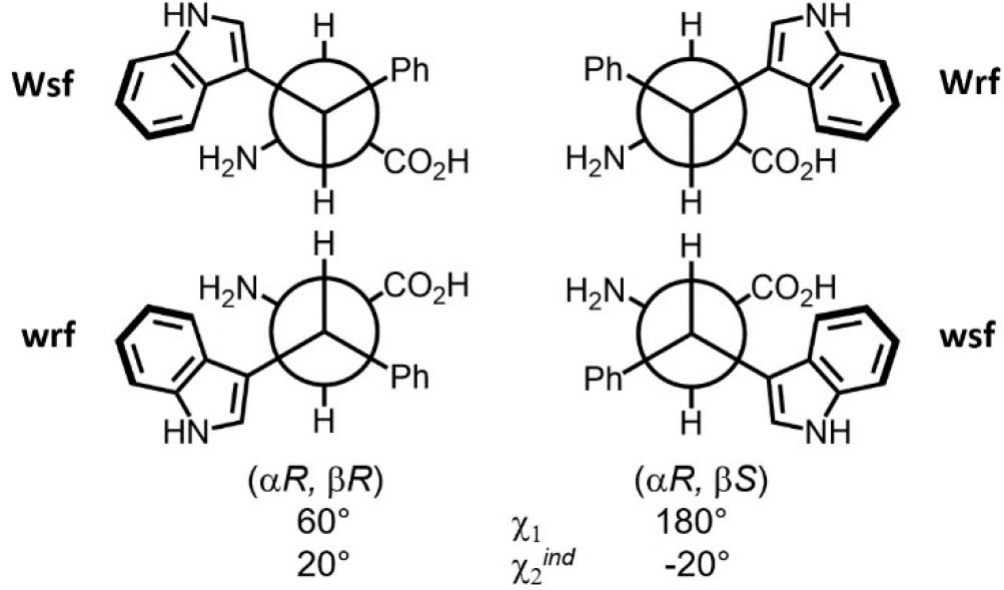
All four stereoisomers of β‐indoyl‐phenylalanine. The side‐chain torsions characterize the d‐configured new building blocks wrf and wsf, which are mirror images of the stereoisomers shown above them.

The conformational properties of the β‐indoylated amino acids become clear when comparing the most active peptide containing encoded amino acids KwFwLL‐NH_2_ (**4**) with the most active peptide containing a β‐indoylated isomer K‐wsf‐FwLL‐NH_2_ (**8**). In both cases, the alternating configurations stabilize an overall cyclic structure with a prominent end‐to‐end NOE contact between w^2^(Hα) and the terminal amide which is even more pronounced in **8** wherein this NOE contact is observed between wsf^2^Hα and the terminal amide. Leu^6^NH has the lowest solvent accessibility, as revealed by the significantly smaller temperature dependence of Δδ Leu^6^NH in both peptides. After performing NMR‐structure calculations the structure ensemble (*vide infra*) with the lowest energy revealed the existence of a Leu^6^NH→Lys^1^CO hydrogen bond spanning a 16‐membered ring of a π‐helix as shown in Figure 4. Another common feature is the strong shielding of Leu^5^γH caused by the w^4^ indole group. Both peptides show remarkably similar NMR spectroscopic signatures described in detail in the Supporting Information. The strong shielding of both Hβ and the H^o,^°^′^ protons of F^3^ in **8** relative to the parent peptide **4** are caused by the indole substituent of wsf. Both phenyl rings rotate fast enough to show AMM′XX′spin systems. ^*3*^ *J*_Hα‐Hβ_ identify a close to equal population of two *χ*1 rotamers of F^3^ in KwFwLL‐NH_2_ which is pushed towards a 70 : 30 ratio in K‐wsf‐FwLL‐NH_2_.^[15]^ Values of ^*3*^ *J*_Hα‐Hβ*proR*_=11.3 Hz and ^*3*^ *J*_Hα‐Hβ*proS*_=5.8 Hz identify an over 80 % *χ*1=180° rotamer of w^4^ in **8**. Thus, wsf^2^ shifts the conformational averaging of the side‐chain rotamers of the both following amino acids F^3^ and w^4^ towards main rotamers. This preferred side‐chain alignment goes in parallel with the overall conformational preference of K‐wsf‐FwLL‐NH_2_ which is characterized by the long‐range NOE contact wsf^2^Hα to one of the terminal amide protons.

**Figure 4:**
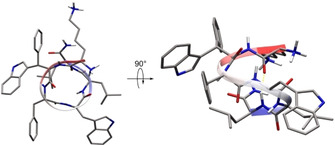
NMR structure ensemble of the β‐indoylated isomer K‐wsf‐FwLL‐NH_2_ (**8**). The minimized structure shows one complete turn of a left‐handed π‐helix. The hydrogen bond from Leu^6^NH to Lys^1^CO stabilizes this conformation and is in agreement with the experimental data. Furthermore, the strong upfield shift of the Leu^5^Hδ^*proS*^ is explained by hydrophobic interaction between the side chains of w^4^ and L^5^.

To characterize the impact of the wsf building block on the conformation of the hexapeptide, energy barrier estimations based on molecular dynamics simulation were performed. As the side chain of wsf is composed of a combination of tryptophan and phenylalanine, these individual amino acids were compared with wsf on a building block level. The amino acid was capped with an acetyl‐group on the N terminus and methylamine group on the C terminus. The resulting structure serves as a model compound with a peptidic character and is often chosen when studying dynamics of amino acids.^[16]^ For better comparison with the literature, simulations of the L‐amino acids were performed. Every model peptide (Ac‐Phe‐NMe, Ac‐Trp‐NMe, and Ac‐Wsf‐NMe) was solvated using TIP3P Water with a salt concentration of 0.15 M. After equilibration, a 500‐ns MD simulation was performed to evaluate the flexibility in the specific side chains. As is visible in Figure 5, the side chain of phenylalanine can freely rotate and perform numerous turns over the simulation period. Since the system exhibits local rotational symmetry about the Cβ‐Cγ‐Cη axis, there are only two distinct orientations accessible, namely −120° and −60° about *χ*2^ph^. In comparison, the larger aromatic residue in tryptophan rotates much slower but still shows multiple transitions during the simulation. Additionally, two conformations can be distinguished at −100° and 100°. The combination of both side chains in wsf increases the rotational barrier for each side chain significantly. Only one rotation about *χ*2 was observed for the phenyl residue after equilibration while the *χ*2^ind^ remains locked in its conformation.

**Figure 5:**
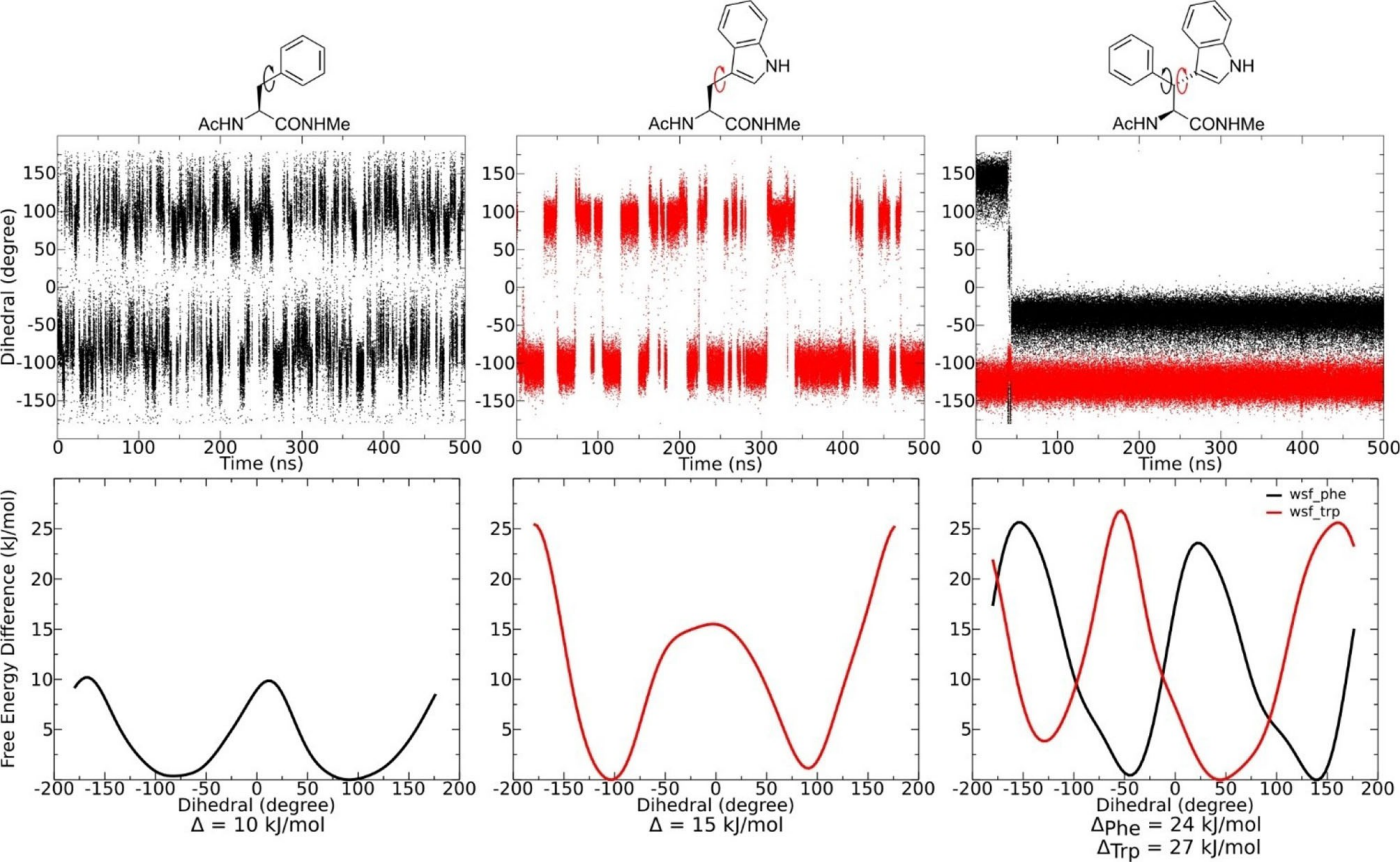
Evolution of the *χ*2 dihedral in Ac‐Phe‐NMe, Ac‐Trp‐NMe and Ac‐Wsf‐NMe. The phenyl side chain can freely rotate and visits different orientations frequently during the simulation. The same behavior is retained in the tryptophan derivate, although less frequent rotations are observed. Both side chains are locked in the wsf derivate and unable to perform any rotations after equilibration. The bottom row shows the free‐energy surface obtained by wt‐metadynamics. A rotation around *χ*2 only requires 10 kJ/mol for Phe but rises to 15 kJ/mol for Trp. A combination of both side chains in Wsf significantly increase the barrier height to 24–27 kJ/mol. For Wsf, two simulations were performed, once biasing *χ*2^ph^ and the second time a bias on *χ*2^ind^ was applied.

In order to be able to more precisely evaluate the energy barriers of the derivatives, well‐tempered (wt) metadynamics simulation were carried out.^[17]^ During wt‐metadynamics the energy basins (constructed by different conformations) visited are filled by adding a positive Gaussian potential. After filling the local minimum, the system is able to transition to another minimum and thus a conformational landscape can be produced. Furthermore, the Gaussian potential can be used to create a free energy estimation of the investigated parameter, in our case the *χ*2 dihedral.

Figure 5 depicts the height of the energy barrier of *χ*2 estimated by wt‐metadynamics. The phenyl residue shows a small symmetric barrier of 10 kJ/mol which is in agreement with the frequent rotations observed in the MD‐simulation. After increasing the side chain to an indole group in tryptophan, the minima are still located at 100 and −100°, while the barrier rises to 15 kJ/mol. Furthermore, the direction of the rotation is predetermined due to the asymmetric shape of the energy profile. The combination of both side chains in Wsf shows the extent of conformational hindrance since the energy barrier increases to 24 kJ/mol for the phenyl and 27 kJ/mol for the indole group. Even though both residues are in proximity, they are still able to rotate independently. During both wt‐metadynamics simulations (either bias on the *χ*2^ph^ or *χ*2^ind^ dihedral), correlated rotations are rare and the molecular gearing effect is less pronounced than expectable from the high barriers about *χ*2^ph^ and *χ*2^ind^. Still, β‐diarylated amino acids with combinations of more hindered aromatic groups are promising building blocks to study the effect of molecular gearing in biomolecular environment and wt‐metadynamics is an efficient method to study this effect.

Finally, to show CH activation of a substituted aliphatic (non‐benzylic) carbon which is representative for most of the amino acids shown in the upper three rows of Figure 1, β‐arylation of Pro was chosen. The arylation of Cbz‐Pro‐8AQ with a Boc‐protected 3‐iodoindole is based on procedures published previously for other aromatic rings.^[18]^ Yet, the large and sensitive indole requires some adjustments of the reaction conditions which are shown in Scheme 2. The β‐indoylation was carried out neat to ensure a high iodoindole concentration. As the iodoindole is temperature sensitive, 80 °C was not exceeded to give the indoylated proline **9** in 54 % yield after six days. The long reaction time is due to the steric demand of the iodoindole as well its temperature sensitivity.

**Scheme 2:**
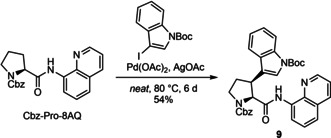
The CH activation of Cbz‐Pro‐8AQ removes Hβ^*proR*^ to yield the β‐indoylated product Cbz‐Wsp‐8AQ. NOEs and ^*3*^ *J* coupling constants identify a strongly preferred pyrrolidine puckering and a single rotamer about the exocyclic bond to the indole substituent.

Wsp is the β‐indoylated α‐amino acid exhibiting the strongest conformational restriction of all amino acids shown in Figure 1. Both methylene groups show significant separation to allow their unequivocal diastereotopic assignment. The pyrrolidine ring assumes a Cγ^*endo*^ pucker which is characterized by a strong ^*3*^ *J_CH_* couplings Cα‐Hγ^*proR*^ and Cβ‐Hδ^*proS*^ (Supporting Information). The strong Hβ‐Hδ^*proR*^ NOE complements the results from the heteronuclear correlation. The relative alignment of indole and pyrrolidine is determined by the characteristic NOE correlations Hϵ^3^‐Hβ and Hδ^1^‐Hγ^*proR*^. This strongly dominating conformer has values of *χ*1=160° and *χ*2=−100°. Other α‐amino acids having β‐methylene groups should react accordingly to further expand the set of β‐indoylated building blocks proposed in Figure 1.

In conclusion, constrained building blocks for bioactive peptides evolved more than 40 years ago in this research field but we are still in the early stages of designing active peptide ligands by combination of synthetic and computational methods. CH activation has contributed fruitfully to this field in the recent years. The bottleneck of removing the directing group of CH activation in the presence of a sensitive side chain like indole was solved by transforming it into a strained and thus reactive amide bond. This synthetic method was streamlined and works on the gram scale as described here for Fmoc‐wsf(Boc)‐OH and Fmoc‐wrf(Boc)‐OH. The complete set of four stereoisomers was investigated in ghrelin receptor inverse agonists. The wide activity range covered by the four hexapeptides (K‐Xaa‐FwLL‐NH_2_ with Wrf^2^, Wsf^2^, wrf^2^, wsf^2^) underlines the importance of the side chain of Trp and identifies the most active, asymmetric β‐diarylated amino acid containing hexapeptide inverse agonist described until now. The alternating configurations of the amino acids in the best inverse agonist stabilize one complete turn of a left‐handed π‐helix. Further studies will show whether the indole groups directly interact with the receptor or indirectly stabilize the receptor bound conformation. The combination of this optimal indole orientation (wsf) and the best 10π aromatic group in β‐(*S*)‐benzothienyl‐D‐Phe is the next systematic optimization step. Rigidified building blocks derived from natural amino acids are a promising tool for the conformational design of bioactive peptides with the aim of constraining the ligands to the bioactive conformation which binds the receptor in a conformational selection event. The 20 β‐indoylated natural amino acids can be expanded to other sets of systematic β‐branched amino acids.

## Conflict of interest

The authors declare no conflict of interest.

## Supporting information

As a service to our authors and readers, this journal provides supporting information supplied by the authors. Such materials are peer reviewed and may be re‐organized for online delivery, but are not copy‐edited or typeset. Technical support issues arising from supporting information (other than missing files) should be addressed to the authors.

Supplementary
