# Supplementary material for: Tryptophan Analogues with Fixed Side‐Chain Orientation: Expanding the Scope
Source: Chembiochem. 2020 Sep 24;22(2):330–5. doi: 10.1002/cbic.202000424 (PMC7891422; doi:10.1002/cbic.202000424)
Supplement: Supplementary file 1 — Supplementary [file CBIC-22-330-s001.pdf]

ChemBioChem

Supporting Information

Tryptophan Analogues with Fixed Side-Chain Orientation: Expanding the Scope

Lennart Nicke, Philip Horx, Ronny Müller, Sylvia Els-Heindl,* and Armin Geyer*

Cbz-Wsp(Boc)-8AQ (**9**) 3.20 g (8.52 mmol, 1.00 eq) of Cbz-Pro-8AQ[1], 14.6 g (42.6 mmol, 5.00 eq) *N-tert*-Butoxycarbonyl-3-iodoindole[2], 0.38 g (1.70 mmol, 20 mol%) $Pd(OAc)_2$ and 3.20 g (19.2 mmol, 1.80 eq) AgOAc were vigorously stirred under inert gas atmosphere at 80 °C for six days. After completion, the mixture was diluted with DCM, filtered through a pad of *celite* and concentrated under reduced pressure. The crude product was purified by column chromatography on silica using toluene/ethyl acetate 5:1 to give 2.72 g (4.60 mmol, 54%) of Cbz-Wsp(Boc)-8AQ as a yellowish solid. Furthermore, 11.5 g (33.6 mmol, 99%) of unused *N-tert*-Butoxycarbonyl-3-iodoindole could be re-isolated as a brown oil. ^{1}H-NMR (500 MHz, 300 K, $CDCl_3$), rotamers, δ = 9.53 (s, 1H), 8.52-8.44 (m, 1H), 8.35-8.28 (m, 1H), 8.15-7.99 (m, 1H), 7.83-7.75 (m, 1H), 7.75-7.69 (m, 1H), 7.47-7.40 (m, 2H), 7.40-7.31 (m, 3.5H), 7.31-7.23 (m, 1H), 7.21-7.14 (m, 2H), 6.99-6.90 (m, 1.5H), 5.33-4.95 (m, 3H), 4.17-4.03 (m, 1H), 4.03-3.89 (m, 1H), 3.74-3.64 (m, 1H), 2.81-2.67 (m, 1H), 2.35-2.26 (m, 1H), 1.46 (s, 9H) ppm; ^{13}C-NMR (125 MHz, 300 K, $CDCl_3$), rotamers, δ = 169.4, 169.1, 155.2, 154.7, 149.3, 147.2, 136.9, 136.6, 135.6, 133.4, 130.1, 128.6, 128.1, 128.0, 127.8, 127.7, 127.6, 127.3, 124.6, 123.5, 123.4, 122.87, 122.81, 121.8, 121.7, 121.1, 119.1, 119.0, 116.9, 115.2, 83.5, 67.3, 65.0, 64.9, 46.5, 46.06, 39.96, 39.1, 28.6, 28.1, 28.0 ppm; HRMS (ESI+): $C_{35}H_{34}N_4O_5Na^+$ [M+Na]+, m/z calcd.: 613.2421; found.: 613.2417.

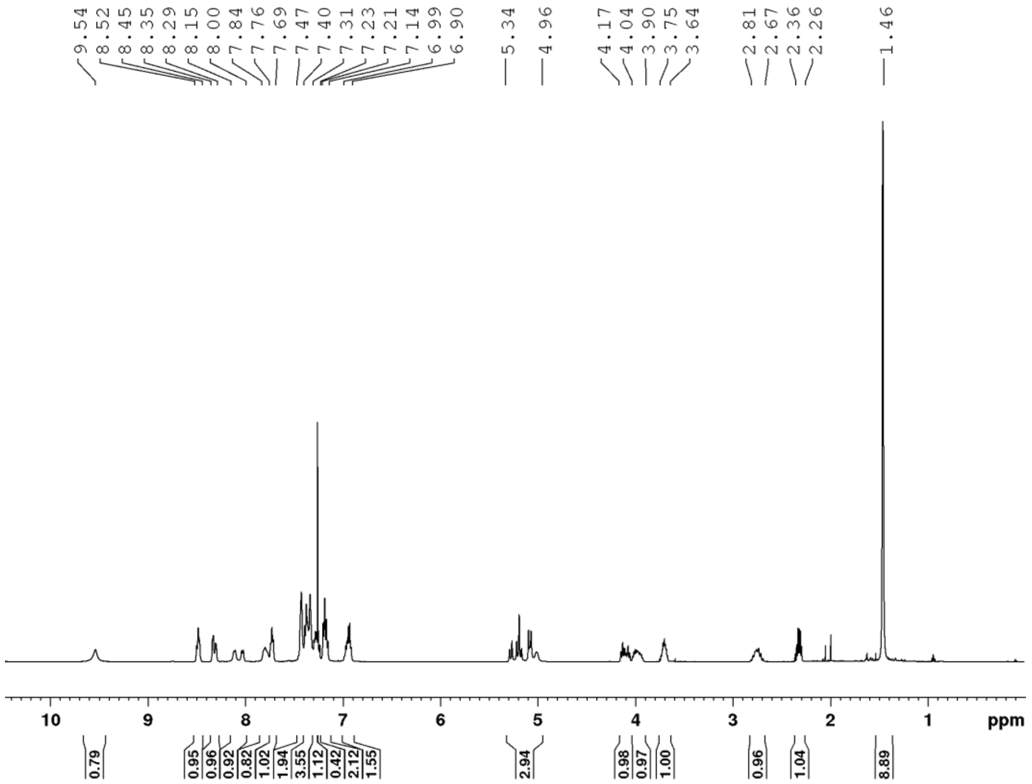

Sup. Figure 1. ^{1}H-NMR (500 MHz, 300 K, $CDCl_3$).

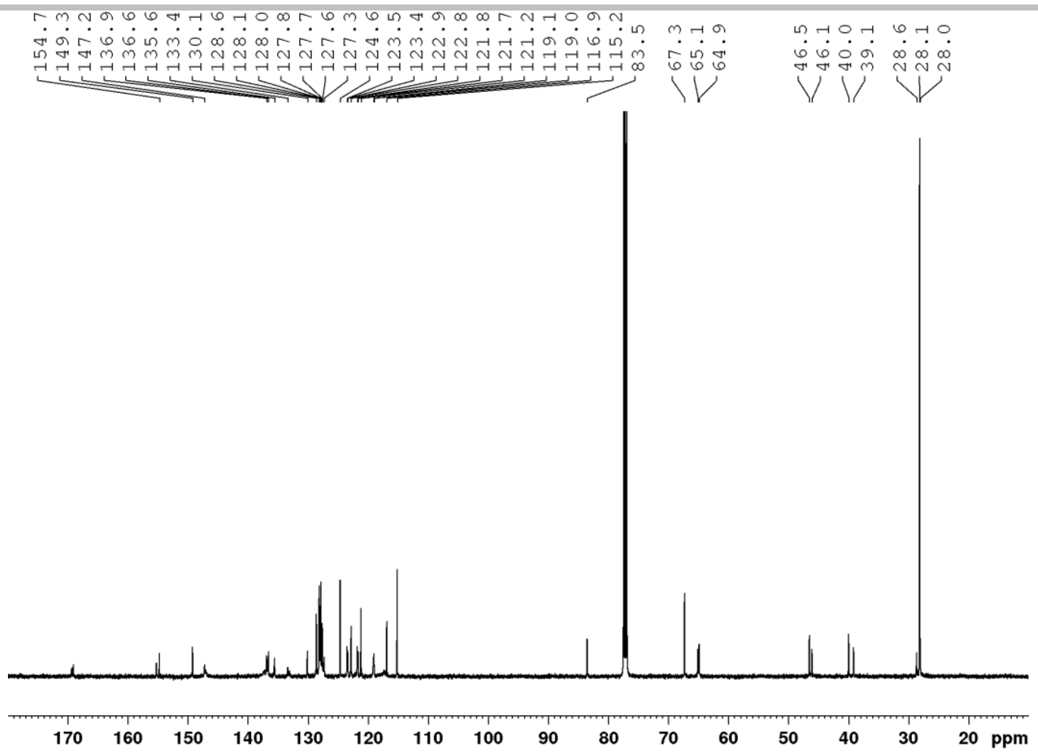

Sup. Figure 2. ^{13}C-NMR (125 MHz, 300 K, $CDCl_3$)

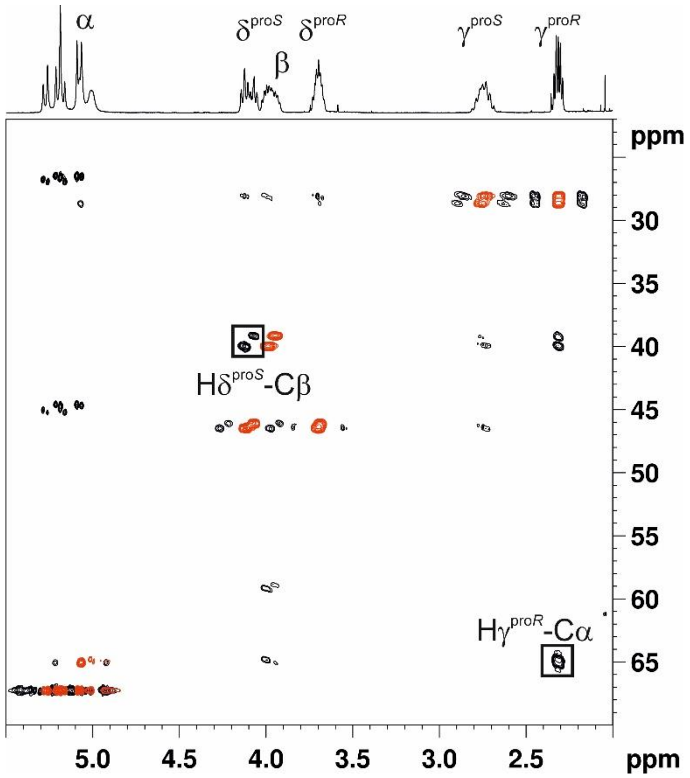

Sup. Figure 3. HMBC spectrum with HSQC-*overlay* in red, 500 MHz, 300 K, $CDCl_3$. Dihedral angle-dependent C-H correlations.

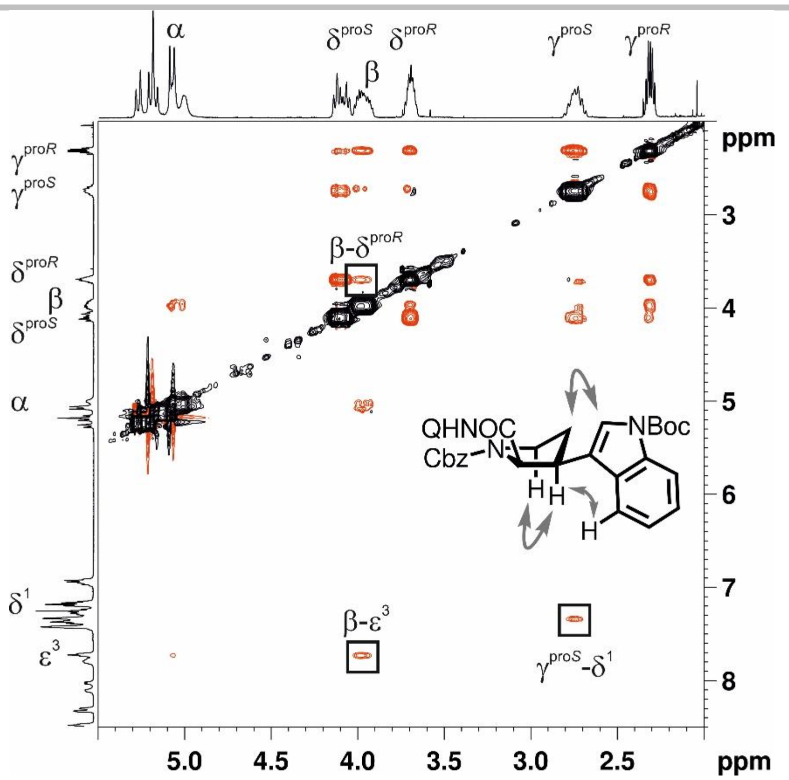

Sup. Figure 4. ROESY spectrum (500 MHz, 300 K, $CDCl_3$). Assignment and conformation.

Peptide synthesis Hexapeptides were synthesized by Fmoc/tBu-solid phase peptide synthesis on TGR RAM resin with an automated multiple peptide synthesizer (SyroI, MultiSynTech, Bochum, Germany) as described previously.[3] β,β-diaryl-amino acids Fmoc-wrf(Boc)-OH and Fmoc-wsf(Boc)-OH were introduced in the peptide with first: 0.5 eq Fmoc-protected amino acid, 0.5 eq DIC and 0.5 eq HOBt in DMF for 4 h and second: 1 eq Fmoc-protected amino acid, 1 eq DIC and 1 eq HOBt in DMF for 16 h. Purity of the peptides was determined by analytical reversed-phase HPLC on at least two of the following columns: Jupiter Proteo (Phenomenex: 250 × 4.6 mm; 4 µm; 90 Å), Kinetex Biphenyl 100 Å (Phenomenex: 250 × 4.6 mm; 5 µm; 100 Å) or Aeris Peptide 100 Å (Phenomenex: 250 × 4.6 mm; 3.6 µm; 100 Å). Peptide identity was analyzed by ESI-Orbitrap-MS (Orbitrap Elite, Thermo Scientific, Waltham, Massachusetts, United States) or MALDI-TOF mass spectrometry (UltraflexIII, Bruker, Bremen, Germany). Observed masses were in full agreement with the calculated masses and peptides with a purity ≥ 95% could be obtained according to analytical RP-HPLC.

Sup. Table 1. Peptide analytics. All compounds were examined towards their identity by ESI-Orbitrap mass spectrometry and purity on two different columns. b = D-3-benzothienyl alanine.

No	Peptide	M_{theo} [Da]	M_{exp} $[M+H]^+$	R_t [%B][a]	R_t [%B][b]	purity [%]
3	KbFwLL-NH_2	907.48	908.49	57.3	47.1	>95
4	KwFwLL-NH_2	890.52	891.53	56.0	44.2	>95
5	K-Wrf-FwLL-NH_2	966.55	967.59[d]	55.0	44.6[c]	>95
6	K-Wsf-FwLL-NH_2	966.55	967.62[d]	54.7	44.3[c]	>95
7	K-wrf-FwLL-NH_2	966.55	967.56	55.7	52.1	>95
8	K-wsf-FwLL-NH_2	966.55	967.56	58.2	49.8	>95

[a] Jupiter Proteo (Phenomenex: 250 × 4.6 mm; 4 µm; 90 Å) | [b] Aeris Peptide 100 Å (Phenomenex: 250 × 4.6 mm; 3.6 µm; 100 Å), if not indicated otherwise | [c] Kinetex Biphenyl 100 Å (Phenomenex: 250 × 4.6 mm; 5 µm; 100 Å) | [d] MALDI-TOF mass spectrometry

IP-One assay COS7 cells stably transfected with the ghrelin receptor fused C-terminally to eYFP were cultured in a humidified atmosphere at 37 °C and 5% CO2 in Dulbecco's modified Eagle's medium with higher glucose supplemented with 10% (v/v) FCS and 0.4 mg/ml hygromycin B. Cisbio IP-One Gq assay kit was used according to previous description.[4] Shortly, 10000 cells/well were seeded out in a 384-well flat white plate and on the next day, stimulation was carried out in triplicates for 3h. 3 µl IP1-d2 and 3 µl Ab-cryptate were added and incubated on a tumbler for 60 min. Fluorescence was measured at 620 nm and 665 nm. HTRF ratio was calculated as the ratio 665/620. Obtained data were analyzed with GraphPad Prism 5.0 (GraphPad Software, San Diego, USA) and normalized to KbFwLL-NH_2. E_{max} is the efficacy of the peptide and represents the difference between constitutive activity and activity at maximal effect of the peptide. EC_{50} is the peptide concentration at half-maximal effect.

NMR data peptide of peptides 4 and 8

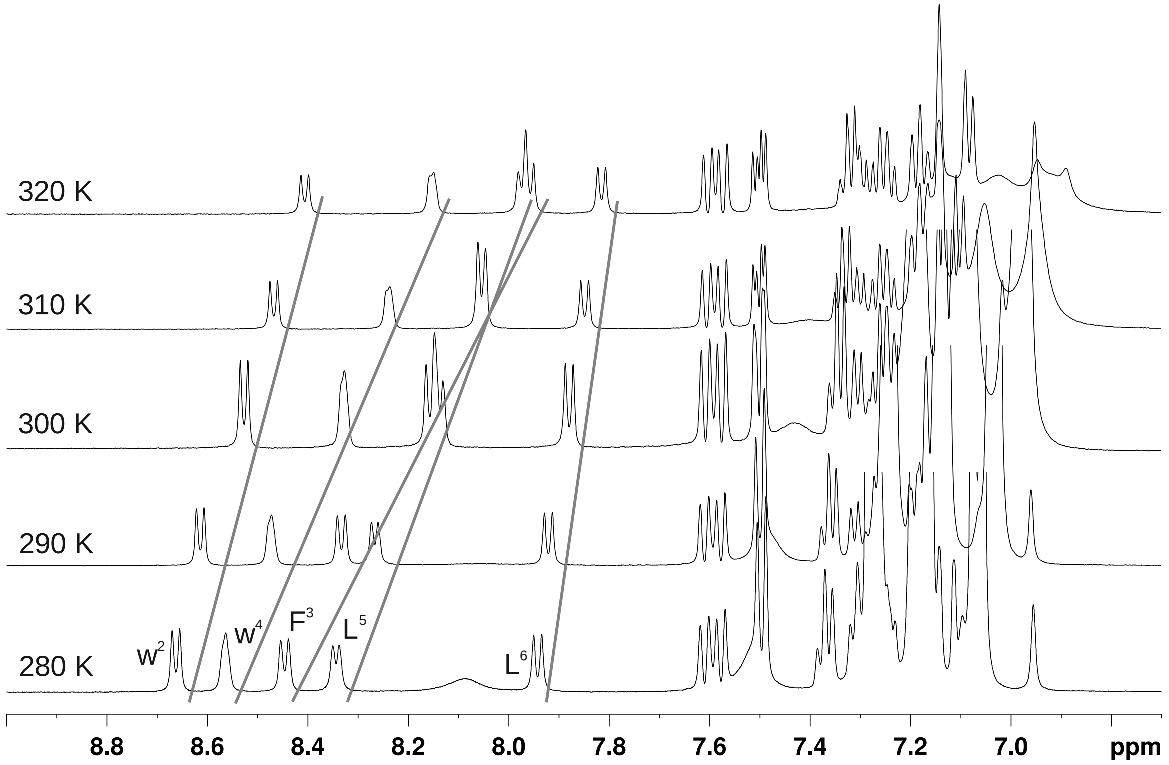

Sup. Figure 5. Peptide KwFwLL-NH_2 (**4**). ^{1}H-NMR spectra, 500 MHz, 50 mM phosphate buffer pH = 3.0/ D_2O 9:1.

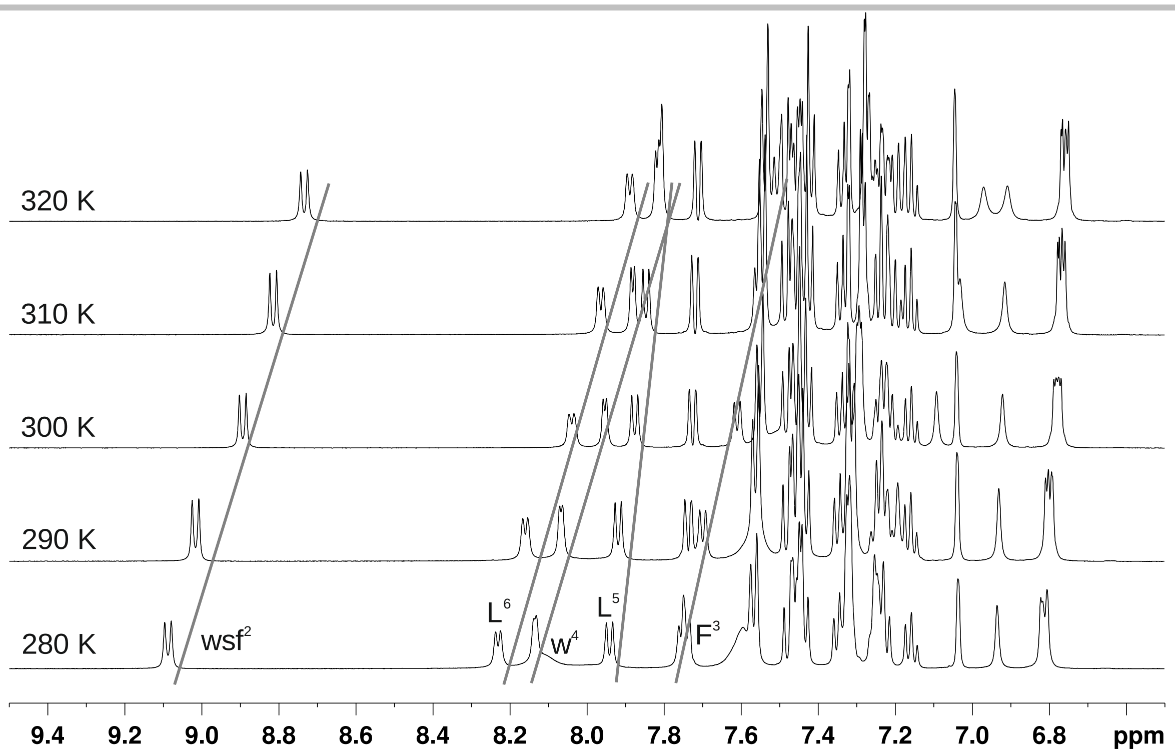

Sup. Figure 6. Peptide K-wsf-FwLL-NH$_2$ (**8**) ^{1}H-NMR spectra, 500 MHz, 50 mM phosphate buffer pH = 3.0/ D$_2$O 9:1.

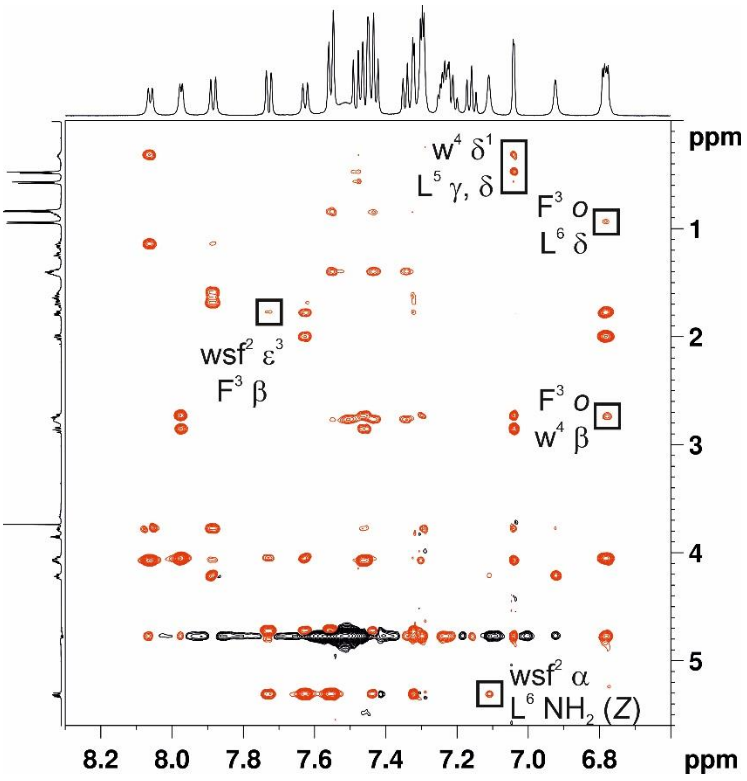

Sup. Figure 7. Peptide K-wsf-FwLL-NH$_2$ (**8**) ROESY spectrum, 600 MHz, 50 mM phosphate buffer pH = 3.0/ D$_2$O 9:1. Structure-determining NOE contacts.

Sup. Table 2. ^{1}H-NMR-temperature dependence $\Delta\delta/\Delta T$ [Hz] of dNH of KwFwLL-NH_2 and K-wsf-FwLL-NH_2 between 280 and 320 K (600 MHz)

Peptide	Xaa^2 NH	Phe^3 NH	trp^4 NH	Leu^5 NH	Leu^6 NH
KwFwLL-NH_2 (4)	-6.4	-12.2	-10.3	-9.3	-3.2
K-wsf-FwLL-NH_2 (8)	-8.9	-6.3	-8.2	-8.6	-3.3

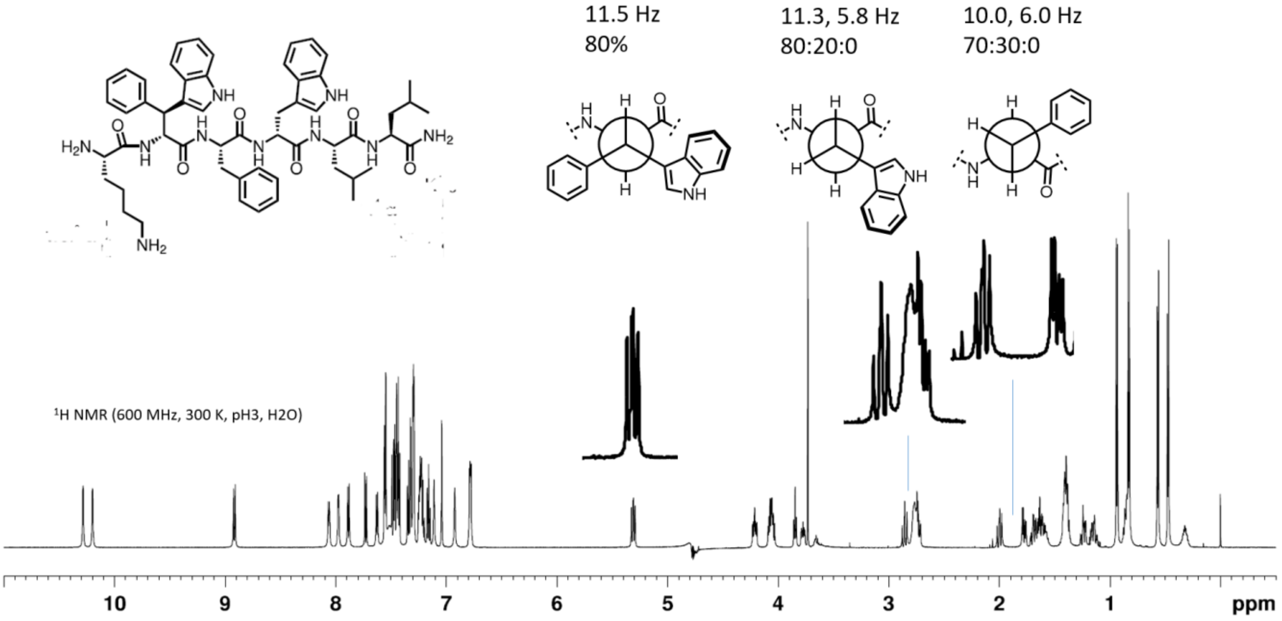

Sup. Figure 8. Side chain rotamers determined according to Lit. 14 (main text) from $^3J_{\alpha,\beta}$ coupling constants after Lorentz-to-Gauss transformation of the ^{1}H NMR (600 MHz).

NMR-Structure Determination The NMR-structure was generated using the Xplor-NIH suite of programs.[5] Distance constraints for the wsf-containing hexapeptide **8** were extracted from ROESY spectra with a mixing time of 80 ms. The cross-peaks were categorized according to their intensities as weak, medium or strong. The coupling constant of amide protons was implemented as a restraint according to the Karplus equation. 1H-NMR measurements at different temperatures revealed that the amide proton of Leu^6 exhibits a small temperature dependency and thus could be involved in a hydrogen bond with a carbonyl oxygen. This additional parameter was evaluated during the refinement of the lowest energy structures and yielded a structure ensemble with no NOE- and dihedral-violations above 0.5 Å. It could be observed that the oxygen of Lys^1 is able to form a hydrogen bond. The calculations started from an extended structure and was heated to 3.500 K and cooled down in 12.5 K steps.[6] After this simulated annealing procedure a short MD-simulation in the eefx2 implicit force field was performed to increase accuracy and quality of the calculated structures.[7]

MD-Simulation All MD-simulations were performed using the GROMACS 2018.4 [8]. The starting structure for the model peptides Ace-Phe-Nme, Ace-Trp-Nme and Ace-Wsf-Nme were prepared using the Xplor-NIH with no restraints. Pdb2gmx program was used to process the pdb files for further simulation. The peptides were solvated respective to system size with 900-1700 TIP3P water molecules in a dodecahedron box.[9] A salt concentration of 0.15 M was added to neutralize the system and mimic the macroscopic salt concentration. A modified version

of the CHARMM36 force field, with additional parameters for the wsf building block included, [10] was used to simulate the system. First an energy minimization was performed for either 500000 steps or until the maximum force reached a value below 50 kJ/mol/nm using a steepest-descent algorithm to remove steric clashes. A 20 ns long equilibration protocol was applied. The first phase was conducted for 10 ns under a NVT ensemble at 300 K using the modified Berendsen thermostat v-rescale [11] with a coupling time step of 0.1 ps to stabilize the temperature of the system. Afterwards, NPT conditions were applied for 10 ns, allowing the pressure to stabilize using a Berendsen barostat with a coupling time step of 2.0 ps. Long range electrostatic interactions were treated using Particle-Mesh Ewald with a short-range cut off of 1.0 nm.[12] In the final production run, which lasted 500 ns, the model peptides were treated without any restrains using the Parrinello-Rahman barostat [13]. After the simulations had finished corrections to the periodic boundary were performed and rotational plus translational motions were removed from the trajectory. For the wt-metadynamics simulation *plumed* 2.4 was used to bias the χ2-dihedral and simultaneously obtain the free-energy profile.[14] A Gaussian with the height of 1.2 kJ/mol with a width of 0.35 was deployed every 1 ps at 300 K.

Free energy estimation conversion One important criterion for the successful performance of a wt-metadynamics simulation is the convergence. A good estimation is the height of the Gaussian deployed during the simulation. As observed in Fig S9, the height of the Gaussian decreases instantaneously which is to be expected for a small system. An additional criterion for the conversion of the wt-metadynamics is the alteration of the free energy surface of the parameter sampled by plumed. The free energy surface changes during the beginning of the simulation but after 200ns only a constant offset is observed. This can be another indication of a converged simulation.

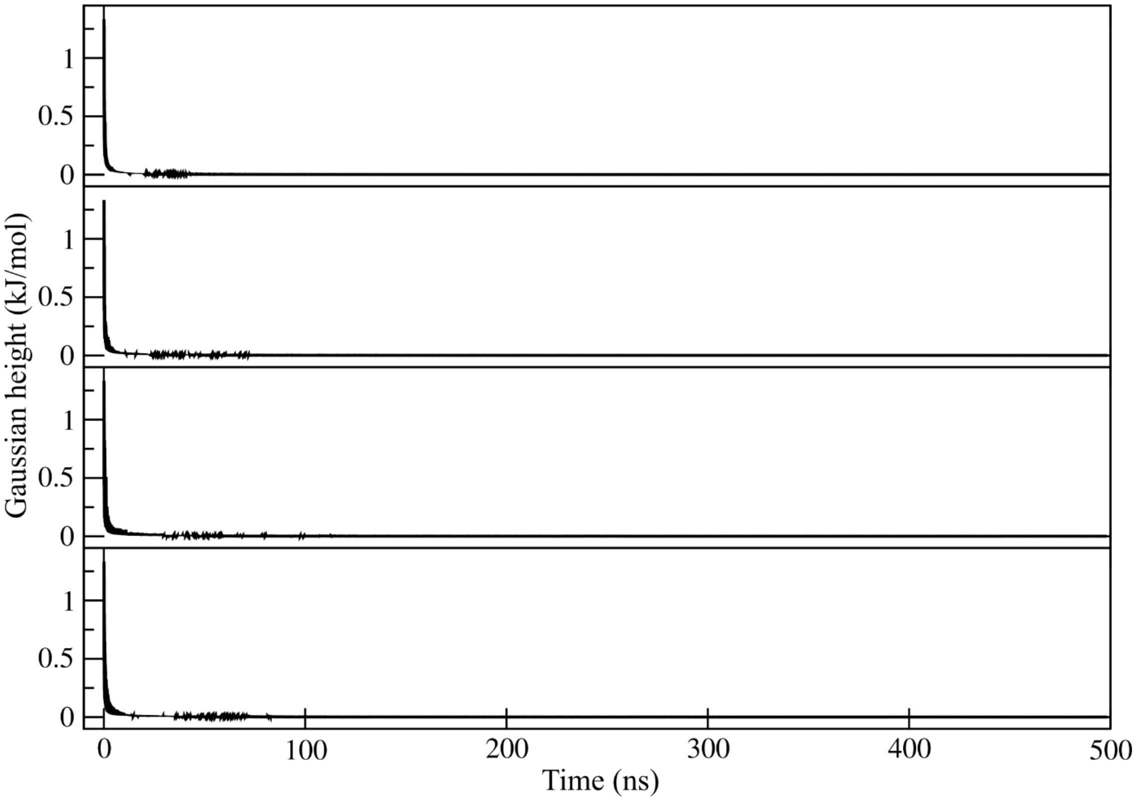

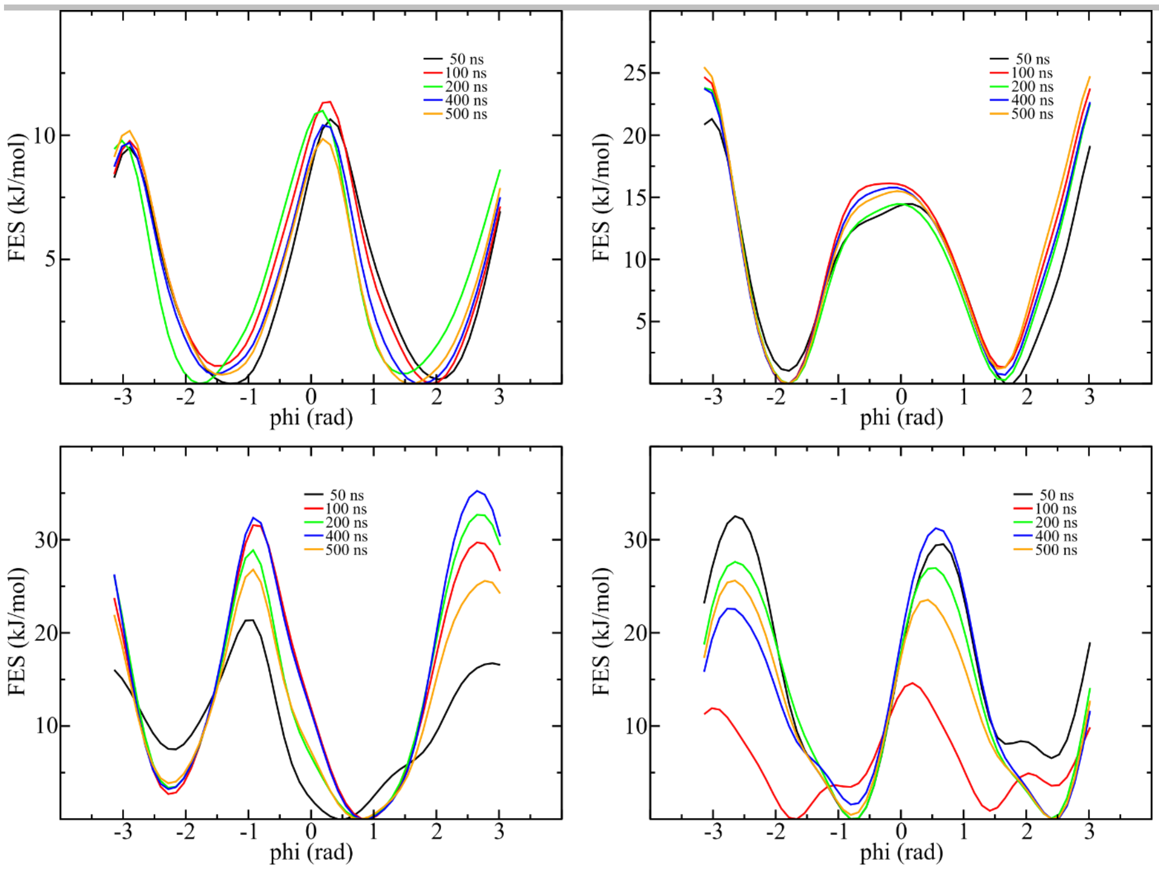

Sup. Figure 9. Metadynamics conversion estimation. On the top the Gaussian height deposition during the wt-metadynamics simulation. On the bottom FES of the biased parameter during the wt-metadynamics at different time points over the simulation. After the initial change of the FES only an offset in the free energy is observed.

Error estimation of the most relevant free energy basins Block analysis was performed to estimate the error of the most relevant free energy basins obtained during the wt-metadynamics. Firstly, the associated weights for each conformation were calculated using an umbrella-sampling reweighting approach, followed by the block analysis. This produces the error for the specific block size. Table S3 list the error of each wt-metadynamics simulation in combination with the respective minimum.

Sup. Table 3. Free energy error estimation of the most relevant free energy basins. A factor of 2.494339 for k_bT was chosen.

Simulated system	Basin (rad)	Free energy error (kJ/mol)
Ace-Phe-NMe	-1.6	0.22
Ace-Trp-NMe	-1.8	0.15
Ace-Wsf-NMe (phe bias)	0.8	0.60
Ace-Wsf-NMe (trp bias)	2.2	0.22

Literature

[1] D. P. Affron, O. A. Davis, J. A. Bull, *Org. Lett.* **2014**, *16*, 4956-4959.

[2] B. O. A. Tasch, D. Antovic, E. Merkul, T. J. J. Müller, *Eur. J. Org. Chem.* **2013**, 4564-4569.

[3] L. Nicke, P. Horx, K. Harms, A. Geyer, *Chem. Sci.* **2019**, *10*, 8634-8641.

[4] L. Nicke, R. Müller, A. Geyer, S. Els-Heindl, *ChemMedChem* **2019**, *14*, 1849-1855.

[5] C. D. Schwieters, J. J. Kuszewski, N. Tjandra, G. M. Clore, *J. Magnet. Res.* **2003**, *160*, 65–73.

[6] E. G. Stein, L. M. Rice, A. T. Brünger, Journal of Magnetic Resonance **1997**, *124*, 154-164.

[7] Y. Tian, C. D. Schweiters, S. J. Opella, F. M. Marassi, *J Biomol NMR*, **2017**, *67*, 35-49.

[8] a) H. J. C. Berendsen, D. van der Spoel, R. van Drunen, Computer Physics Communications **1995**, 91, 43–56; M. J. Abraham, T. Murtola, R. Schulz, S. Páll, J. C. Smith, B. Hess, E. Lindahl, SoftwareX **2015**, 1–2, 19–25; c) S. Páll, M. J. Abraham, C. Kutzner, B. Hess, E. Lindahl, in Solving Software Challenges for Exascale (Eds.: S. Markidis, E. Laure), Springer International Publishing, **2015**, pp. 3–27.

[9] W. L. Jorgensen, J. Chandrasekhar, J. D. Madura, R. W. Impey, M. L. Klein, *J. Chem. Phys.* **1983**, *79*, 926–935.

[10] J. Huang, S. Rauscher, G. Nawrocki, T. Ran, M. Feig, B. L. de Groot, H. Grubmüller, A. D. MacKerell, *Nature Methods*, **2017**, *14*, 71–73.

[11] G. Bussi, D. Donadio, M. Parrinello, *J. Chem. Phys.* **2007**, *126*, 014101

[12] T. Darden, D. York, L. Pedersen, *J. Chem. Phys.* **1993**, *98*, 10089–10092.

[13] a) M. Parrinello, A. Rahman, Phys. Rev. Lett. **1980**, *45*, 1196–1199; b) M. Parrinello, A. Rahman, Journal of Applied Physics **1981**, *52*, 7182–7190.

[14] G. A. Tribello, M. Bonomi, D. Branduardi, C. Camilloni, G. Bussi, *Computer Physics Communications*, **2014**, *185*, 604–613.
